# The Therapeutic Effects of Ketamine in Mental Health Disorders: A Narrative Review

**DOI:** 10.7759/cureus.23647

**Published:** 2022-03-30

**Authors:** Carolina Sepulveda Ramos, Matthew Thornburg, Kelly Long, Kiran Sharma, Julia Roth, Diana Lacatusu, Reece Whitaker, Daniel Pacciulli, Sulma Moredo Loo, Mohammad Manzoor, Yun-Yee Tsang, Sydney Molenaar, Karthikeyan Sundar, Robin J Jacobs

**Affiliations:** 1 Dr. Kiran C. Patel College of Osteopathic Medicine, Nova Southeastern University, Fort Lauderdale, USA; 2 Medical and Behavioral Research; Health Informatics; Medical Education, Nova Southeastern University, Fort Lauderdale, USA

**Keywords:** suicide ideation, depression, non-competitive n-methyl-d-aspartate receptor antagonist, ect, ptsd, therapeutic effects, alcohol, cocaine, ketamine

## Abstract

Ketamine, a non-competitive N-methyl-d-aspartate receptor antagonist, is commonly used as an anesthetic and analgesic but has recently shown promising research in treating certain psychiatric conditions such as depression, post-traumatic stress disorder (PTSD), suicidal ideation, and substance use disorder. Due to its euphoric, dissociative, and hallucinogenic properties, ketamine has been abused as a recreational drug, which has led to rigid regulation of medication. The COVID-19 pandemic has been an unprecedented challenge for the American population which was reflected in increased reports of problems regarding their mental health. Mood disorders have dramatically increased in the past two years. Approximately one in ten people stated that they had started or increased substance use because of the COVID-19 pandemic. Furthermore, rates of suicidal ideation have significantly increased when compared to pre-pandemic levels, with more than twice the number of adults surveyed in 2018 indicating suicidal thoughts “within the last 30 days” at the time they were surveyed. Moreover, many responders indicated they had symptoms of PTSD. The PubMed database was searched using the keyword “ketamine,” in conjunction with “depression,” “suicidal ideation,” “substance use disorder,” and “post-traumatic stress disorder.” The inclusion criteria encompassed articles from 2017 to 2022 published in the English language that addressed the relationship between ketamine and mental health disorders. With this sharp increase in the prevalence of psychiatric disorders and an increased public interest in mental health combined with the promise of the therapeutic value of ketamine for certain mental health conditions, including suicidal ideation, this narrative review sought to identify recently published studies that describe the therapeutic uses of ketamine for mental health. Results of this review indicate that ketamine’s therapeutic effects offer a potential alternative treatment for depression, suicidal ideation, substance use disorders, and PTSD.

## Introduction and background

Ketamine, a non-competitive N-methyl-d-aspartate (NMDA) receptor antagonist, is commonly used as an anesthetic and analgesic, but has recently shown promising research in treating certain mental health conditions such as depression, schizophrenia, substance use disorder (SUD), post-traumatic stress disorder (PTSD), and suicidal ideation [1,2]. Due to its euphoric, dissociative, and hallucinogenic properties, ketamine has been abused as a recreational drug, which has led to rigid regulation of the pharmaceutical [3]. However, the increased rates of mental health disorders (e.g., mood disorders, PTSD) and public interest in mental health have elicited further consideration for its treatment applications, making this the opportune time for the research and development of new treatment modalities to address these diagnoses [4]. Due to the dearth of evidence regarding the therapeutic value of ketamine for certain mental health conditions, including suicidal ideation, more research to address the major gaps is warranted. The purpose of this narrative review is to identify recently published studies that describe the therapeutic uses of ketamine for mental health.

## Review

The PubMed database was searched using the phrase “ketamine AND depression OR suicidal ideation OR ECT OR substance use disorder OR cocaine addiction OR alcohol abuse OR post-traumatic stress disorder” and filtered for studies published between 2017 and 2022, including randomized controlled trials, meta-analysis, systematic reviews, and reviews published in the English language. The initial search yielded 416 results. The 416 articles were evaluated and eliminated based on their relevance to the relationship between ketamine and the mental health disorders listed above. The exclusion criteria ruled out animal studies, pediatric studies, case studies, and studies that did not focus on ketamine’s use. After thoroughly screening for appropriateness and relevance, 48 articles were included in this review.

Depression

The Diagnostic and Statistical Manual of Mental Disorders (DSM-V) defines major depressive disorder (MDD) as five or more symptoms occurring for at least two weeks. These symptoms must include depressed mood or loss of interest in activities but can also include weight changes, fatigue, and thoughts of suicide [5]. In 2019, the Center for Disease Control and Prevention (CDC) found that 4.7% of adults over the age of 18 had regular feelings of depression, while 10.8% of patients had depression indicated on their medical records [6,7]. The first line of pharmacologic treatment for MDD is typical antidepressants, which include selective serotonin reuptake inhibitors (SSRIs), selective norepinephrine reuptake inhibitors, and tricyclic antidepressants. However, these medications routinely take several weeks to take effect and come with a variety of side effects including weight gain and sexual dysfunction, resulting in adherence issues [8]. In addition, many those with depression do not gain the desired benefit (i.e., decreased depression) after taking anti-depressant medication. 

While treatment-resistant depression (TRD) is not officially defined in the DSM-V, it is considered two prior treatment failures of an antidepressant regimen using the adequate dosage and duration of the medication in those with MDD [9]. An estimated 30.9% of those with MDD can be defined as having TRD [10]. In those with TRD, electroconvulsive therapy (ECT) is the gold standard therapeutic method. ECT consists of electrically stimulating the brain while the patient is under general anesthesia. While ECT has shown to be efficacious in those with MDD, it has numerous adverse effects such as amnesia, headache, and disorientation [11]. It has been postulated that ketamine, due to its anesthetic and anti-depressive effects, could be the ideal anesthetic for use in ECT. However, at this time there is a lack of sufficient evidence that demonstrates that ECT in conjunction with ketamine as an anesthetic is more efficacious than using typical anesthetics during ECT to treat depression [12,13]. Alternatively, ketamine alone has been shown to have an antidepressant effect within 40 minutes of administration with a single intravenous (IV) infusion in those with MDD, with maximum efficacy occurring at 24 hours post-infusion. However, this beneficial effect of ketamine on depression has been shown to disappear one to two weeks after initial treatment [14].

Due to the stigmatization of ketamine as a street drug, it was previously administered via IV to avoid use outside of a hospital setting. However, esketamine nasal spray has recently been approved by the FDA for the treatment of TRD [15]. Esketamine is the (S)-enantiomer of ketamine, which has a stronger binding affinity for the NMDA receptor than its (R)-enantiomer counterpart [16]. The IV preparation of ketamine most widely used is in its racemic (R,S)-enantiomer form [16]. Studies have shown that esketamine combined with an antidepressant resulted in lower depression rates than a placebo nasal spray [17-19]. Because of esketamine’s ease of use, it can be self-administered by patients outside of a clinical setting. However, studies have shown that the infusion of ketamine is more effective than the esketamine nasal spray. This may be because early studies have demonstrated that (R)-ketamine is possibly more efficacious and longer acting in treating patients with depression than (S)-ketamine [20]. Thus, in serious circumstances, patients should receive IV ketamine whenever possible [21].

Suicidal ideation

There is no universal definition for suicidal ideation, but it is typically defined as thoughts and contemplations about death and suicide [22]. Suicide is another pervasive problem in the United States, with 45,979 Americans dying by suicide in 2020 [23]. In addition to its use in patients with depression, ketamine has been theorized to help patients with acute suicidal ideation. An exploratory analysis by Grunebaum and colleagues showed that the odds of suicidal ideation with a score of 0 on day 1 were 2.8-fold greater for the ketamine group, although the difference only neared statistical significance (p=0.08) [1]. Among the study participants who continued to have suicidal ideation on day 1, no differential drug effect was seen on planning suicide but greater improvement was seen in the ketamine group in suicidal desire and ideation (estimate=1.37, df=58, t=2.02, p=0.049) [1]. IV and intranasal ketamine were each shown to have reductions in suicidal ideation within 24 hours of administration [1]. In addition to treating those with TRD, ECT is also used to treat those with acute suicidal ideation [24]. When ECT’s efficacy was compared to that of ketamine, ketamine was shown to have higher decreases in suicidal ideation after 24 hours versus one week (three treatments) of ECT therapy [24]. While the beneficial effects of ketamine further increased a week after infusion, ECT therapy demonstrated similar results after six treatments (two weeks) and eventually outperformed ketamine for treatment of suicidal ideation after a mean of 7.5 treatments [24]. It is hypothesized that the concurrent use of ketamine and ECT may have antagonistic effects with one another. In ECT regimens that use barbiturates, such as thiopental as an anesthetic, the anti-seizure properties of the barbiturate are theorized to interfere with treatment [22].

In treating suicidal ideation, it may be beneficial to have patients start ketamine therapy and then wait a period of time to begin ECT therapy rather than using the two methods concurrently. With an initial rapid infusion of ketamine, patients would be able to receive more immediate treatment for their acute feelings of suicidal ideation that could prevent suicides that may otherwise be addressed too slowly. However, because ketamine’s antidepressant effects are shown to disappear one to two weeks after infusion, having the patient then start ECT therapy would be imperative in preventing the recurrence of suicidal ideation after the therapeutic effects of ketamine have subsided [14]. Giving adequate time between ketamine administration and the beginning of ECT could prevent the possible antagonistic effects of using the two therapies concurrently [25]. It is suggested that a time period of five days after the infusion of ketamine may be the appropriate time to start ECT, but the safety and efficacy of different treatment regimens would need to be studied.

A current study, ELEKT-D, is underway to attempt to compare the efficacy of ketamine and ECT in those with TRD. Hopefully, the ELECKT-D study can shed some light on the efficacy of ketamine compared to ECT in those with TRD [26]. A recent study has shown that ketamine can cause a decrease in acute suicidal ideation for at least six weeks when used as an adjunct therapy with anti-depressant therapy [1]. The researchers hypothesize that this increased length of efficacy is due to the participants remaining on their antidepressants during this study. Ketamine’s duration of efficacy for treatment of acute suicidal ideation should be further researched before more concrete conclusions can be made. Additionally, intranasal esketamine, has shown to significantly increase resolution for suicide risk 24 hours after the first dose [18]. Having an easily useable ketamine for acute suicide risk may be crucial for those that cannot reach the hospital or other treatment facility.

Substance use disorder

According to the American Psychiatric Association, SUD is a condition in which there is frequent use of a substance despite its potentially fatal or life-threatening effects [27]. SUD applies to the use of a drug or chemical substance [28]. These can range from illicit street drugs to alcohol.

SUD tends to occur in patients who have concomitant mental health disorders, such as patients with schizophrenia, patients suffering from depression [29,30]. Furthermore, the scope of the disorder extends beyond the reaches of just a neurochemical issue. SUD is debilitating to the patient’s social life, finances, and community [28]. Prevention and treatment may not be completely effective for some individuals [31]. Yet, new treatments are currently being developed like the novel course of treatment that is seen with ketamine.

It has been found that there is a greater frequency of cocaine use associated with lifetime alcohol dependence [32]. Ketamine has shown efficacy in prolonging abstinence in individuals with SUD as well as showing reduced intake and cravings of cocaine users [33]. This is important as addictive substances act under a reinforcing mechanism where dopamine signaling is propagated in the nucleus accumbens [34]. Chronic exposure to the addictive substance then leads to glutamatergic ridden neuroplastic changes [34]. It is thought that ketamine may be of possible help in addiction medicine by enhancing neuroplasticity and neurogenesis and disrupting relevant functional neural networks created through addiction [33].

Alcohol tends to bind to multiple dopamine receptors non-specifically within the nucleus accumbens, leading to symptoms of addiction [35]. Traditional treatments to alcoholism have included: cognitive behavioral therapy, motivational interviewing, and pharmaceuticals such as disulfiram or naltrexone [36,37]. It was found in a study conducted in 2019, that cognitive behavioral therapy held a statistically significant impact on alcohol consumption in early intervention, but not late [38]. This can be a problem as the goal of therapy is to prevent relapse long term. For ketamine specifically, one study found that a single infusion improved likelihood of abstinence, time to relapse, and decreased the likelihood of heavy drinking, in persons with alcohol use disorder [39]. However, notable limitations within the study included the necessity of a larger cohort study to provide more accurate and reliable data that may be better generalized and a longer study duration as participants were only monitored for 21 days post-infusion [39]. Thus, a study using a larger, less homogenous cohort, followed for a longer period of time, is warranted for more accurate results. More research is needed to compare the efficacy of older alcoholic treatments to that of ketamine.

Alternatively, cocaine’s mechanism of addiction causes an increase in astrocytic calcium events that lead to the creation of synapses within the nucleus accumbens [40,41]. The treatment of choice for cocaine addiction includes psychosocial therapy with an emphasis on cognitive-behavioral therapy [39]. Of note, there is not a major stress on pharmaceutical applications to aid with cocaine addiction, as there has been with alcohol addiction [36,37]. Yet, ketamine has been found to have beneficial effects in these patients. In a trial conducted in 2019, it was found that 48.2% of individuals treated with ketamine-maintained abstinence from cocaine use over the last two weeks of the trial, compared with 10.7% of the subjects in a midazolam control group [42]. The study concluded that a single ketamine infusion improved the outcome of cocaine-dependent adults engaged in mindfulness-based behavioral modification, by promoting abstinence, diminishing craving, and reducing the risk of relapse [42]. However, the sample in the study was relatively homogeneous, with a majority of subjects being African American and male, with minimal psychiatric comorbidity [42]. Thus, the effects of early-life ketamine administration maybe age and sex-specific, and it is possible that adolescent administration of ketamine for the treatment of depression may lead to an increased risk of addiction later on in life [42]. Further research must be conducted on human subjects in order to determine the significance of these results in a clinical setting.

Post-traumatic Stress Disorder

PTSD is defined as a mental health disorder that meets the following clinical criteria: exposure to a traumatic event, presence of intrusive symptoms related to the event, persistent avoidance of triggering stimuli, negative alterations to cognition and mood, and marked alterations in arousal and reactivity [43]. For diagnostic purposes, the symptoms must be present for more than a month after the traumatic event first occurs [43]. Given the heightened sense of stress and worry introduced by COVID-19, the pandemic has been associated with an increase in both triggering stimuli and rates of PTSD [44,45]. The only pharmacological treatments approved for PTSD by the FDA are SSRIs [46]. However, SSRIs are used as a maintenance therapy, not a means of remission [46]. As a result, there has been a search for alternative methods to treat PTSD, either as a remission treatment or a maintenance treatment.

In a retrospective study assessing peritraumatic use of ketamine in war-wounded soldiers, it was concluded that ketamine did not affect PTSD prevalence [47]. Although ketamine neither decreases nor increases the chances of developing PTSD, it does have a potential use for maintenance treatment [48-50]. Despite the suggestion of the Veterans Affairs guidelines to not use ketamine in PTSD due to the lack of current evidence in favor of its use, some patients with PTSD have received off-label treatment with ketamine [48,49]. In 2020, a retrospective study analyzed internet posts on the Bluelight website, a public forum intended to provide a platform for people to discuss their drug use, and evaluated the self-reported use of ketamine in patients with PTSD [49]. The results found that patients with self-perceived PTSD had decreased incidence of negative symptoms when using ketamine [49]. Although the study was unable to identify the validity of the anonymous statements, the conjecture was that people felt ketamine’s dissociative effects led to a state of no arousal, allowing them to avoid over-aroused states [49].

Clinical trials were conducted in a regulated manner that demonstrated similar findings to those suggested by the Bluelight website retrospective study [50,51]. One study evaluated the efficacy of ketamine use in the treatment of chronic PTSD, showing promising results, with prompt reduction in intrusion, avoidance, and negative mood and cognition symptoms 24 hours after initial treatment and throughout a two-week treatment period when compared to the use of midazolam [49]. Additionally, greater numbers of participants showed responsiveness to ketamine when compared to that of midazolam, resulting in a number needed to treat (or number of patients that need to be given the medication to see a benefit) of 2.1 [51]. Concomitant depression symptoms showed similar outcomes, with a significant decrease in symptoms recorded 24 hours after each infusion over a two-week period [50,51]. Remission rates across PTSD and associated TRD were 80% and 93.3%, respectively [50]. Both inpatient psychiatric hospital days and admission rates post ketamine treatment further supported these results, demonstrating a greater than 65% reduction in rates of both [52]. 

Hesitancy surrounding the use of ketamine in treatment primarily involves the potential for adverse effects. However, studies suggest these effects, if present at all, are typically mild and resolve relatively quickly [51-53]. On the evaluation of cognitive decline two hours post ketamine infusion, results demonstrated: decreased attention, executive function, and verbal memory, but sparing of working memory and a complete return to baseline performance within 24 hours [53]. While short-term dissociative effects were reported in some participants immediately post administration, these effects may be more likely to occur with repeated infusions [50-52]. Physiologic side effects experienced include lightheadedness or dizziness, sedation, fatigue, nausea, and vomiting, but frequency of these effects varied by study [51,52]. No hypertensive crises, psychotic crises, or bladder toxicity was reported [52]. Less consistent were results regarding loss of response and potential for relapse, with some studies suggesting loss of response can occur quickly, often within six weeks [50,51]. Contrastingly, other results showed no tolerance development despite long treatment periods, with decreased doses to achieve similar efficacy over time [52]. Conflicting results such as these are additional proof that while ketamine does appear to have a promising future in the treatment of PTSD, further research is warranted regarding ideal dosages, routes of administration, and duration of treatment course in order to make well-supported recommendations for optimal outcomes.

## Conclusions

Ketamine’s therapeutic effects offer a potential alternative treatment for depression, suicidal ideation, SUD, and PTSD. Ketamine has shown to be an effective treatment in rapidly decreasing depressive symptoms in those with depression. However, more studies should be done to determine ketamine’s role in the inpatient setting and its effectiveness in those with other psychiatric comorbidities such as anxiety and bipolar disorder. Ketamine may likely play a larger role in treating those with depression now that esketamine, a nasal form of administration, has been approved by the FDA. Additionally, the use of ketamine and ECT together in treating suicidal ideation is an interesting new direction to take these two treatments. Ketamine’s short-acting effects and ECT’s longer-acting benefits may allow the treatments to better help patients combat their suicidal ideation, albeit in distinct roles of therapy. However, more studies must be performed on the efficacy of using these two treatments together. In terms of SUD, ketamine has shown promising treatment results by decreasing relapse events, increasing abstinence, and diminishing cravings. However, more research is needed to compare the efficacy of traditional alcoholic treatments to that of ketamine. Further research must be conducted on human subjects in order to determine the significance of ketamine treatments on cocaine addiction. In PTSD, conflicting study outcomes are proof that while ketamine does appear to have a promising future in its treatment, further research is warranted regarding ideal dosages, routes of administration, and duration of treatment course in order to make well-supported recommendations for optimal results.
